# Antifibrotic Drugs against Idiopathic Pulmonary Fibrosis and Pulmonary Fibrosis Induced by COVID-19: Therapeutic Approaches and Potential Diagnostic Biomarkers

**DOI:** 10.3390/ijms25031562

**Published:** 2024-01-26

**Authors:** Aurelio Perez-Favila, Idalia Garza-Veloz, Lucia del Socorro Hernandez-Marquez, Edgar Fernando Gutierrez-Vela, Virginia Flores-Morales, Margarita L. Martinez-Fierro

**Affiliations:** Doctorado en Ciencias con Orientación en Medicina Molecular, Unidad Académica de Medicina Humana y CS, Universidad Autónoma de Zacatecas, Zacatecas 98160, Mexico; chaure7@gmail.com (A.P.-F.); idaliagv@uaz.edu.mx (I.G.-V.); lucia.hdz@uaz.edu.mx (L.d.S.H.-M.); fer_drgtz@yahoo.com.mx (E.F.G.-V.); virginia.flores@uaz.edu.mx (V.F.-M.)

**Keywords:** antifibrotic drugs, pulmonary fibrosis, COVID-19, biomarkers

## Abstract

The COVID-19 pandemic has had a significant impact on the health and economy of the global population. Even after recovery from the disease, post-COVID-19 symptoms, such as pulmonary fibrosis, continue to be a concern. This narrative review aims to address pulmonary fibrosis (PF) from various perspectives, including the fibrotic mechanisms involved in idiopathic and COVID-19-induced pulmonary fibrosis. On the other hand, we also discuss the current therapeutic drugs in use, as well as those undergoing clinical or preclinical evaluation. Additionally, this article will address various biomarkers with usefulness for PF prediction, diagnosis, treatment, prognosis, and severity assessment in order to provide better treatment strategies for patients with this disease.

## 1. Introduction

Coronaviruses are a family of viruses that primarily target the human respiratory system. Historically, coronaviruses have affected humans, the most recent being severe acute respiratory syndrome (SARS-CoV) and Middle East Respiratory Syndrome (MERS-CoV), all of which pose a significant threat to public health worldwide [1]. Following the report of the first cases of a respiratory syndrome of unknown etiology in Wuhan City, Hubei Province (31 December 2019), authorities identified a novel coronavirus (SARS-CoV-2) that causes the clinical disease called coronavirus disease 19 (COVID-19) [2]. SARS-CoV-2 is a single-stranded RNA virus and has a size of 29,891 nucleotides. It also has several structural proteins: Spike (S), Envelope (E), Membrane (M), and Nucleocapsid (N) [3]. Globally, according to World Health Organization (WHO) data as of 28 December 2023, there were 773,119,173 million confirmed cases of COVID-19, including 6,990,067 deaths. In Mexico to the same date, 7,702,731 confirmed cases of COVID-19 and 334,947 deaths were reported [4,5].

### 1.1. Viral Transmission and Clinical Features of COVID-19 Disease

The SARS-CoV-2 virus can spread through small droplets or aerosols [6]. Sick individuals expel these particles when speaking, coughing, or sneezing. The particles can travel up to 1 m in the air before settling on environmental surfaces or the mucosa of nearby individuals [7]. The first step of SARS-CoV-2 viral infection is the recognition and binding of the receptor in host cells, then fusion with the cell membrane follows. The virus has been reported to target pulmonary epithelial cells, whereby transmission of SARS-CoV-2 occurs by binding the S protein receptor-binding domain with angiotensin-converting enzyme 2 (ACE2) receptors [8,9]. When the virus infects the host cell, its RNA genetic material is released into the cytoplasm. This process is known as uncoating [10]. Within the cytoplasm, RNA is transcribed to produce non-structural proteins (nsps) and replicate polyproteins, including RNA polymerase and RNA-dependent helicase. These form the RdRp complex, which is responsible for the transcription and replication of viral RNA. Subsequently, the viral structural proteins are translated. The proteins S, N, M, and E are processed in the membrane of the rough endoplasmic reticulum (ER) and then transported to the ER-Golgi intermediate compartment (ER-GIC), where they assemble with RNA to form viral particles. The final step is the release of the virions from the cell, in a process known as exocytosis [10,11]. The symptoms of COVID-19 are variable. The most common symptoms include fever, cough, fatigue, and headache. In severe cases, dyspnea and chest tightness may occur, which can lead to death [12,13]. The incubation period for SARS-CoV-2 is 6–7 days, while the time between illness onset and doctor visit is 4–5 days [13]. The mortality rate of the disease caused by the SARS-CoV-2 virus is lower than that of the aforementioned coronaviruses, with a fatality rate of 2–3% [14].

### 1.2. Post-COVID-19 Syndrome

People who manage to overcome SARS-CoV-2 infection usually present with a series of persistent symptoms; it was then that the terms prolonged COVID-19, post-COVID-19 syndrome, or long-term COVID-19 began to be used by the medical and scientific community to describe the symptoms that occur after week 12 of having started with the first symptoms [15,16]. According to various reports, the most commonly reported symptoms after COVID-19 include fatigue, dyspnea, chest pain, headache, cough, and hair loss. Some of these symptoms are associated with lung disease, such as cough, chest discomfort, decreased lung-diffusing capacity, sleep apnea, and pulmonary fibrosis (PF) [17,18]. The proportion of COVID-19 patients who remain asymptomatic is generally low. Two studies of COVID-19 survivors reported that only between 10.8% and 18.6% of patients were symptom-free after recovering from the disease [19,20]. All these reports reinforce that most of the symptoms present after post-COVID-19 infection are related to lung problems.

A meta-analysis recently revealed that one-third of individuals experience persistent fatigue, and more than one-fifth of individuals experience cognitive impairment for 12 or more weeks after being diagnosed with SARS-CoV-2. Furthermore, there is a correlation between acute illness severity, hospitalization, or longer hospital stays and post-COVID-19 symptoms or reduced quality of life [21]. Another study found that during post-COVID-19 pulmonary evaluations, the diffusing capacity for carbon monoxide (DL_CO_) was below 80%. Specifically, 29% of patients in the group with a scale of 4 (admitted to the hospital but requiring supplemental oxygen) and 56% of patients in the groups with scales of 5–6 (admitted to the hospital requiring high-flow nasal cannula (HFNC) and admitted to the hospital requiring extracorporeal membrane oxygenation) had DL_CO_ below 80%. Additionally, after six months, most patients reported experiencing at least one symptom, with fatigue and muscle weakness being the most common, along with anxiety and depression [22].

## 2. Overview of the Respiratory System

The lungs are vital organs of the respiratory system and are responsible for exchanging gases between the environment and the bloodstream. They play a crucial role in respiration [23].

**Figure 1:**
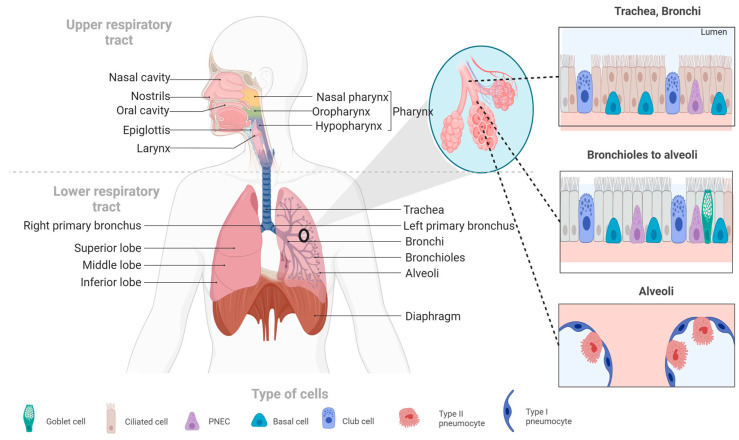
The human respiratory system. This figure illustrates the main components of the human respiratory system, including the upper and lower respiratory tracts, as well as the cell types that make up the respiratory system’s tissues.

The respiratory system is divided into the respiratory tract and lung parenchyma. The airways are formed by the bronchus, which bifurcates into the trachea and then into bronchioles and alveoli (see Figure 1). The parenchyma is responsible for gas exchange [24]. Breathing involves inhaling oxygen from the environment, which then diffuses through the bloodstream via systemic circulation to produce adenosine triphosphate (ATP), the energy source for our cells. As a byproduct, we exhale carbon dioxide (CO_2_) [25]. The respiratory process consists of two basic stages: inspiration and expiration. The volume of air that enters and leaves the lungs during this process is known as tidal volume [26].

The lungs can develop various pathologies that can be divided into: (1) obstructive pulmonary disease, in which there is an alteration in breathing out and includes asthma and chronic obstructive pulmonary disease (COPD) [27]; (2) restrictive lung disease, in which the lung expansion is compromised and causes a decrease in pulmonary volumes; notably, the total lung capacity (TLC) is reduced. Some examples of restrictive lung disease are idiopathic PF (IPF), sarcoidosis, and pneumoconiosis [23,28].

## 3. Pulmonary Fibrosis (PF)

Interstitial lung disease (ILD) and PF are a group of lung diseases that share an inflammatory component and fibrosis of the lung parenchyma [29]. ILD has several attributed causes, including environmental exposures, microbial agents, occupational hazards, drug exposure, and autoimmune or connective tissue diseases [30]. One specific type of ILD is idiopathic pulmonary fibrosis (IPF), which is the most common type due to its unknown cause and poor prognosis [29]. IPF may be very similar to post-COVID-19 or induced PF by COVID-19 in terms of profibrotic pathophysiological mechanisms and response to treatments [31]; therefore, the term used in this review, PF, refers to any pulmonary fibrotic condition [32].

**Figure 2:**
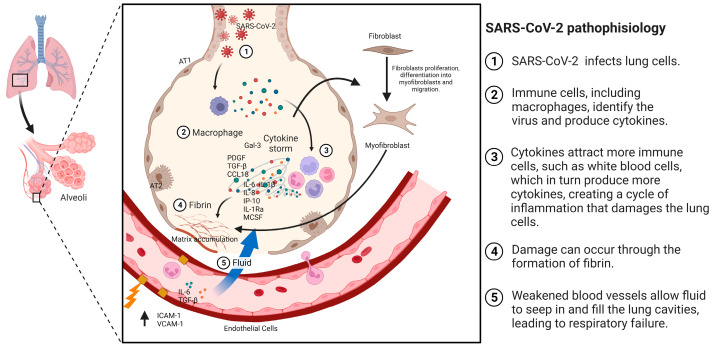
Pathophysiology of PF associated with COVID-19. The mechanism of pulmonary fibrosis (PF) begins with an injury or the entry of SARS-CoV-2. This triggers the activation of macrophages and the cytokine storm phenomenon, which, in turn, activates other cells, such as fibroblasts. These fibroblasts differentiate into myofibroblasts, leading to the accumulation of extracellular matrix and the development of fibrosis.

Patients with acute respiratory distress syndrome (ARDS) and pneumonia have a higher likelihood of developing pulmonary fibrosis (PF), with rates ranging from 20% to 50%, respectively. Among patients with COVID-19, 40% develop ARDS, and of those cases, 20% are severe [33]. PF is recognized as a sequela of ARDS, characterized by failure of alveolar re-epithelialization, activation of fibroblasts, and excessive deposition of collagen and other extracellular matrix (ECM) components that alter normal lung architecture [34]. The exact mechanism by which PF occurs is not completely defined; however, studies suggest that some cytokines, especially transforming growth factor β (TGF-β) and cells such as fibroblasts and myofibroblasts, in addition to angiotensin-converting enzyme 2 (ACE2), play a role in this process [35]. PF is a consequence of a severe attack on the alveolar wall of the lung [36], which is produced by the deregulation of one or more stages in the wound healing process: injury, inflammation, and repair. Here, inflammation is an essential factor in the development of PF [37]. SARS-CoV-2 can act as the initiator of alveolar wall injury (Figure 2) [38]. Fibroblasts are effector cells that participate in fibroproliferation, migrating to the site of injury by stimulating fibroblast growth factor (FGF), platelet-derived growth factor (PDGF), TGF-β, and cytokines. In addition, fibroblast proliferation and differentiation to myofibroblasts are stimulated by endothelial growth factor (VEGF), PDGF, TGF-β, and interleukin-1 (IL-1) [39].

### 3.1. Risk Factors for PF Associated with COVID-19

The development of PF associated with COVID-19 includes risk factors directly attributable to the patient, such as age, gender, alcohol or tobacco use, the presence of comorbidities, and genetic susceptibility. Other factors specific to the disease include the severity of the illness, the need for ICU services, mechanical ventilation, and the presence of ARDS [40,41].

#### 3.1.1. Age and Sex

Previous reports suggest that PF is more common in men than in women, with approximately 70% of patients who develop PF being male [41]. Men are also more likely to die from COVID-19 (OR = 1.39; 95% CI: 1.31–1.47) [42]. The age of onset of PF is approximately 65 years; advanced age has been associated with the risk of developing PF (r = 0.574; *p* = 0.01) [34]. It has been reported that a reduction of 10% in telomere length with age was associated with a 1.35-fold increase in PF development [43].

#### 3.1.2. Smoking and Alcoholism

Smoking has been associated with various lung diseases, such as cancer, pulmonary emphysema, and pulmonary fibrosis [44]. An increased risk of progression of PF in patients with COVID-19 has also been documented (OR = 14.5, 95% CI: 1.6–25.0) [45]. Alcohol consumption is an aggravating factor in the progression of PF and a factor associated with increased odds of ARDS (OR = 1.89; 95% CI: 1.45–2.48) [46].

#### 3.1.3. Comorbidities

The presence of comorbidities such as diabetes mellitus (DM) and pulmonary and cardiovascular diseases is considered a risk factor for the development of PF [40]. As an example, patients with DM (a disease that affects various organs, mainly the cardiovascular system and kidneys) [47] had a 50% increased risk of developing PF [48].

#### 3.1.4. Mechanical Ventilation and ICU Length of Stay

Patients who are admitted to the ICU and require invasive mechanical ventilation have a higher risk of developing PF [49]; compared to patients who did not require mechanical ventilation, 72% of individuals on mechanical ventilation had fibrotic abnormalities [43]. Similarly, in patients discharged after COVID-19 infection, mechanical ventilation increased the risk of PF by 4.45 (OR = 4.45; 95% CI: 1.27–15.58) [49].

#### 3.1.5. Acute Respiratory Distress Syndrome

In COVID-19 patients who develop ARDS, 20% were reported to develop PF [50]. Similarly, in a follow-up study of 27 patients receiving mechanical ventilation for ARDS, after extubation, 85% of patients developed PF, and a relationship to the duration of pressure-controlled inverse-ratio ventilation was observed (*p* < 0.001) [34].

## 4. Mechanisms Involved in the Development of IPF

Fibroblasts synthesize collagen, fibronectin, and ECM [51]. Myofibroblasts—other cells involved in fibrosis—secrete factors such as VEGF and TGF-β, produce denser but more disorganized ECM than fibroblasts, and persist longer at the injury site [52]. One cytokine involved in tissue repair is TGF-β. Sources of TGF-β include platelet granules and macrophages. TGF-β is predominantly expressed in PF and helps stimulate the formation of ECM, collagen, fibronectin, elastic fibers, and matrix substances (Figure 2) [53].

### 4.1. Cells Involved in the Development of IPF

Several cells contribute to the development of PF by producing growth factors and cytokines, which contribute to the fibrotic process and pathogenesis of the disease.

#### 4.1.1. Macrophage Activation

Macrophages are a type of cell that plays an essential role in innate immunity and inflammation [54]. There are two main macrophage phenotypes: M1, which is associated with proinflammatory responses, and M2 macrophages (M2a, M2b, M2c, and M2d), which play crucial roles in anti-inflammatory processes [55]. The transition from one phenotype to another is highly variable, and activation can be a rapid and reversible process [56]. Macrophage activation occurs through several pathways. The canonical IRF/STAT signaling pathways are activated by IFN and TLR to activate the M1 macrophage phenotype (STAT1) or to activate the M2 phenotype. IL-4 and IL-3 (STAT6) are required [57]. M1 macrophages increase the regulation of IRF5, thereby stimulating cytokines (IL-12, IL-23, TNF) involved in Th1 and Th17 responses [58]. On the other hand, it has been seen that interleukin IL-10 can activate the expression of genes mediated by STAT3, which can be associated with the M2 phenotype [59]. 

Two populations of macrophages have been identified in the regulation of pulmonary homeostasis: alveolar macrophages (AMs), which are located in the lumen of the airways, whose markers are CD11b^low^, CD11c^++^, and CD169^+^, and interstitial macrophages (IMs), which are located in the lung parenchyma, with markers CD11b^+^, CD11c^low^, CD169 [60]. Macrophage signaling pathways in PF are TGF-β/Smad, Wnt/β-catenin, and interleukin signaling [61,62,63]. In a study conducted with patients with severe COVID-19, the accumulation of CD163^+^ macrophages were reported, some of which co-expressed the chemokine receptor CXCR3 and complement factor C1Q. In addition, collagen deposition was reported very prominently, for which the authors propose that SARS-CoV-2 promotes genetic programs associated with fibrosis in macrophages [64].

#### 4.1.2. Activation of Fibroblasts and its Differentiation to Myofibroblasts

Fibroblasts are mesenchymal cells whose function is homeostasis and production of ECM [65]. In the lung, fibroblasts are found in the ECM of the interstitial space, where they participate in early and late wound repair [66]. For the progression of PF, a key factor is the proliferation and differentiation of fibroblasts into myofibroblasts [67]. TGF-β 1 participates in stimulating the differentiation of fibroblasts into myofibroblasts [68]. A study found that miR-21 expression is involved in collagen production by fibroblasts or fibroblast-like cells through negative regulation of Smad7. This suggests that Smad7 could be a potential therapeutic target in PF [69].

**Figure 3:**
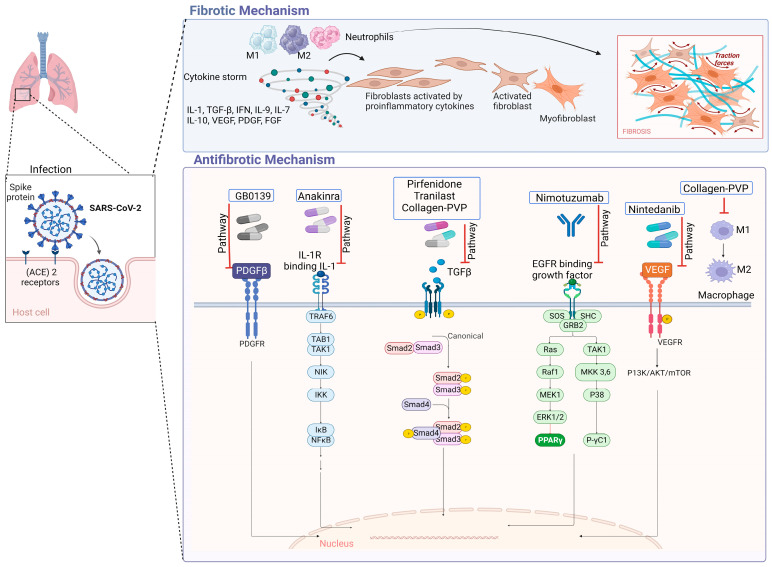
Antifibrotic drugs. Figure shows a possible antifibrotic mechanism involved in the treatment of pulmonary fibrosis induced by COVID-19. Pirfenidone, Nintedanib, and collagen-polyvinylpyrrolidone have different mechanisms that may regulate PF, while Nimotuzumab, Anakinra, Tranilast, and GB0139 have more specific mechanisms of action [75,76,77,78,79,80,81].

In IPF, type 1 collagen is the most abundant and relevant collagen, as it is deposited in greater quantities in the ECM [70,71]. Fibroblasts and myofibroblasts mainly secrete this type of collagen due to the action of several cytokines and factors, such as TGF-β, in addition to the hypoxia pathway [72]. In a study conducted by Philp, C. J et al., the inhibition of the formation of crosslinks through transglutaminase 2 modified the behavior of fibroblasts by reducing adhesion and proliferation of these; it also improved the renewal of ECM, which would mean that it could be a potential therapy for IPF by reducing excess ECM at the site of injury [73]. Figure 3 displays antifibrotic drugs with evidence of their use in PF related to COVID-19 and their possible mechanisms of antifibrotic action. For instance, Nimotuzumab inhibits EGFR, thereby inhibiting the JAK/STAT pathway and preventing fibrosis from progressing or occurring [74].

### 4.2. Signaling Pathways Involved in the Development of IPF

The pathological process by which IPF develops is very complex and involves the activation of alveolar epithelial cells as well as the proliferation of fibroblasts and differentiation into myofibroblasts; likewise, various growth factors such as TGF-β intervene, leading to irreversible damage to the alveolar architecture.

#### 4.2.1. TGF-β Signaling

TGF-β is a member of a family of polypeptides that modulate various cellular functions, the most important of which are cell proliferation, differentiation, and apoptosis [82]. There are three major inactive isoforms of TGF-β (TGF-β1, TGF-β2, TGF-β3), which are synthesized and secreted in association with latency-associated peptide (LAP) [83]. When there are normal conditions, TGF-β in its inactive conformation binds to LAP, which, through disulfide bonds, binds to latent TGF-β-binding proteins, crosslinked with the ECM [84]. TGF-β can be activated within the large latent complex (CLL) in various ways, for example, acidification, temperature variations, oxidation, proteolytic cleavage, or through interaction with integrins, which will cause TGF-β to interact with TGF-β receptors (TGF-βR), and the consequent action is to mediate downstream effects [85]. TGF-β plays an important role in the development of IPF [86,87]. Upregulation and activation of all TGF-β isoforms and receptors have been reported to be involved in the pathogenesis of IPF [88].

It is postulated that TGF-β signaling, which is a profibrotic inducer, is stimulated by the protein N of SARS-CoV. Since this protein is 90% similar to that of SARS-CoV-2, it is considered to be one of the possible mechanisms of pulmonary fibrosis associated with SARS-CoV-2 infection [89]. Another possible mechanism is that coronaviruses can induce the lowering of ACE2; therefore, there is a reduction of angiotensin II (Ang II) in the lungs. In this way, Ang II could upregulate TGF-β and connective tissue growth factor [47]. Once the lung injury occurs, TGF-β, in addition to being released by the injured epithelial and endothelial cells, is released by macrophages, fibroblasts, T cells, and monocytes, which results in a self-sustained process [85]. The canonical TGF-β signaling pathway will involve the activation of SMAD proteins that will activate transcription [90]. TGF-β, in its non-canonical pathway, can act on mitogen-activated protein kinase (MAPK), extracellular signal-regulated kinase (ERK), and N-terminal Jun N kinase (JNK), as well as RHO-associated kinase (ROCK) and AKT pathways [91]. Some of these pathways are involved in the induction of the epithelial–mesenchymal transition (EMT), which promotes the exacerbation of fibrotic lesions [92].

#### 4.2.2. Activation of Integrins

Integrins belong to a family of proteins composed of one of 18 unique α subunits and eight β subunits that pair to create one of the 24 identified integrins [93]. They play a crucial role in maintaining cell–cell junctions, cell–EM adhesion, and signal transduction from the ECM to the intracellular compartment [94]. Integrins have been found to participate in various disease processes, including sarcoidosis and PF [95]. Recent research has demonstrated that the S protein of SARS-CoV-2 binds to integrin ⍺5β1 in vascular endothelial cells (ECs) through the RGD domain. This binding activates the expression of nuclear factor kappa β (NF-κβ), which is responsible for vascular leakage and leukocyte adhesion. These findings suggest a new mechanism by which SARS-CoV-2 affects ECs and identify integrin ⍺5β1 as a potential therapeutic target for treating COVID-19-related inflammation [96]. αVβ6 is another integrin that recognizes RGD (Arg-Gly-Asp). It is overexpressed in many aggressive epithelial tumors, as well as in pulmonary and hepatic fibrosis [97]. A study found that high levels of αVβ6 suggest the presence of fibroblast foci and can also identify a specific endotype of progressive fibrotic lung disease [98].

### 4.3. Growth Factors Involved in Idiopathic Pulmonary Fibrosis

Several growth factors, including PDGF, CTGF, and FGF, have been reported to play a role in the development of IPF by regulating the activity of fibroblasts.

- PDGF is a mitogenic molecule that affects various connective tissue cells and other cell types [99]. This factor is composed of polypeptide chains A and B linked by a disulfide bond, which can form homo- and heterodimers [100]. PDGF is produced by several cell types, including macrophages, platelets, endothelial cells, and fibroblasts [101]. It has four isoforms that bind to two receptor tyrosine kinases (PDGFR α and β). These receptors are expressed in higher quantities in cells such as fibroblasts and myofibroblasts, where they participate in survival, proliferation, and migration [102]. PDGF works together with TGF-β to promote the release of activated alveolar macrophages and epithelial cells, which are directly involved in the self-activation cycle of fibrosis [103]. High levels of PDGF have been associated with FP, both in lung tissue and in bronchoalveolar lavage fluid, as demonstrated in various animal studies with models of PF [104]. PDGF-A and PDGF-C have also been found to be overexpressed in various cell types in a mouse model of injured lungs [105]. During pulmonary fibrosis, alveolar macrophages promote the release of PDGF, which contributes to the proliferation of alveolar myofibroblasts and fibrogenesis [106].
- Fibroblast growth factors (FGFs) belong to a family of 22 members [107,108]. These can be divided into hormone-like FGFs (FGF 15/19, 21 and 23), canonical FGFs (FGF 1–10, 16–18, 20 and 22), and intracellular FGFs (FGF 11–14) [109]. FGFs are involved in several biological responses, interacting with heparan sulfate glycosaminoglycans (HPSGs). Released FGFs interact with cell surface receptor (FGFR) domains [110]; a complex of FGF, FGFR, and HPSG is formed, and thus, signaling is carried out [111]. Abnormal FGF signaling is implicated in various pathologies and is known to participate in the pathogenesis of PF [112]. The inhibition of FGF signaling by using FGFR2c decreased lung fibroblast proliferation and differentiation in vitro and in vivo in a murine model [113]. The alteration in FGFR1 and FGF1 signaling is critical for fibroblast migration in PF [114]. In SARS-CoV infection, the EGFR signaling pathway remains active even after the virus is eliminated. This pathway is believed to be responsible for the effect of FGF in the conduction to PF [115].
- Connective tissue growth factor (CTGF) belongs to the IGFBP family, which is known as insulin-like growth factor-binding protein 8 (IGFBP-8) [116,117]. This 38 kDa mitogenic peptide is involved in fibrotic processes and stimulates migration, fibroblast proliferation, and ECM production [118]. CTGF is a cytokine that participates in the activation of fibroblasts; it was observed that in alveolar epithelial cells, the expression levels of CTGF were drastically reduced by inhibiting the Rho pathway. Additionally, the epithelial cells of the lung in the mice indicators of CTGF increased the activity of the promoter of CTGF when the authors treated them with bleomycin, an inducer of PF [119]. CTGF can interact with other molecules to develop its fibrotic effects, thus contributing to the generation of fibrosis; among the molecules with which it interacts, there are cytokines and growth factors such as IGF1, BMP4, BMP7, TGF-β and VEGF. This interaction with other molecules may positively or negatively alter the signaling pathways of which they are participants, resulting in changes in cell adhesion, migration, differentiation, angiogenesis, myofibroblast activation, deposition, and remodeling of ECM, which causes changes in structure and alters tissue remodeling [120].
- Metalloproteinases (MMPs) belong to the family of extracellular endopeptidases, whose primary function is the degradation of ECM components, while tissue inhibitors of metalloproteinases (TIMPs) block the activity of MMPs [121]. The participation of proinflammatory cytokines affects the overexpression of MMPs, which increases its activity and thus participates in the remodeling of the airways [122]. The level of TIMPs is increased in the presence of a fibrotic process such as PF [123]. MMP-2 and MMP-9 are downregulated in severe COVID-19 patients [124]. MMP-2 has shown a correlation with mortality in patients with COVID-19, so it could be a potential prognostic predictor for patients with COVID-19 [125]. One study reported that MMP-7 correlates with clinical and functional predictors of disease severity and mortality, so it can be used to distinguish IPF from other chronic diseases [126].

## 5. Antifibrotic Treatments and Evidence Level

Currently, there is no drug available to cure FP. The treatment guidelines for PF (an official ATS/ERS/JRS/ALAT Clinical Practice Guideline) recommend a systemic approach that includes treating comorbidities, oxygen therapy, and pulmonary rehabilitation [127]. Although several drugs have been developed to expand the therapeutic options, Table 1 presents only those drugs that have shown favorable results in treating IPF and its association with COVID-19. These are similar to pirfenidone and nintedanib, which are FDA-approved for IPF treatment [128]. However, in studies of PF associated with COVID-19, in a case report of a 66-year-old patient with PF, pirfenidone showed favorable results: FVC values improved from 1.98 L to 2.30 L, total lung capacity (TLC) improved from 61.7% predicted to 72.3%, and carbon monoxide diffusing capacity (DL_CO_) improved from 30.3% predicted to 47.9% [129]. On the other hand, nintedanib was evaluated in 42 patients with COVID-19 pneumonia and was found to help improve the SpO_2_/FiO_2_ ratio (144.38  ±  118.05 vs. 55.67  ±  75.09, *p* = 0.006) [75].

Other drugs with encouraging results include nimotuzumab, which was studied in 41 patients (31 severe and 10 moderate COVID-19), and it was found that nimotuzumab reduces IL-6 and PAI-1 through the EGFR pathway, which inhibits tyrosine kinase activity and may prevent fibrosis in patients with severe and moderate COVID-19 at high risk of worsening [76].

Other drugs have been used in PF and, due to their mechanisms of action, could be scaled for use in PF associated with COVID-19. These drugs are listed in Table 2. Some of these molecules are in phase II, including Pamrevlumab (FG-3019), which attenuated the progression of IPF and was well tolerated. Drugs in the preclinical stage include EW-7197, which was evaluated in a murine model of male C57BL/6 mice and Sprague-Dawley rats. It was shown to be a therapeutic agent against liver, kidney, and lung fibrosis by inhibiting TGF-β-/Smad2/3 and ROS signaling [132]. Similarly, Alamandine was evaluated in a murine model of bleomycin-induced PF in 28 Wistar rats and showed favorable results in collagen deposition and improved pulmonary ventilatory mechanics [133]. AT13387 was evaluated in a study of male C57Bl/6J mice inoculated intratracheally with 15 mg/kg of the drug. AT13387 was found to reduce alveolar inflammation, fibrosis, collagen deposition, and the development of chronic lung injury and airway hyper-responsiveness [134]. Another molecule, Piceatannol (PIC), was evaluated in a C57BL/6 mouse model of bleomycin-induced PF and was shown to attenuate bleomycin-induced collagen deposition and myofibroblast accumulation [135].

Due to the complex nature of treating PF in the context of COVID-19, scientists worldwide are proposing new molecules daily to expand the therapeutic options for this disease. Currently, several ambitious projects have been registered in this regard. Table 3 displays the studies of these drugs that have a ClinicalTrials.gov registry (consultation date: November 2023) for the therapeutic approach of IPF. These studies are in different stages, either recruitment or completed with results. For instance, the study ‘Treatment of PF Due to COVID-19 With Fuzheng Huayu’ has been completed, and the drug Fuzheng Huayu Tablet was evaluated in phase II. Another study, ‘Pirfenidone compared to placebo in post-COVID19 pulmonary fibrosis’, is still in the recruitment stage. These trials can be categorized based on their current study phase: 3 (NA Phase), 3 (Phase 1), 2 (Phase 1–2), 6 (Phase 2), 2 (Phase 2–3), 1 (Phase 3), and finally 4 (Phase 4), with Phase 2 being the most common.

## 6. Biomarkers Associated with IPF, Its Progression, and Its Possible Application in PF Associated with COVID-19

The process of establishing a diagnosis of IPF requires a multidisciplinary medical team of pathologists, pulmonologists, and radiologists. First, other known causes of interstitial lung disease (ILD) are ruled out, and then a computed axial tomography scan is performed to look for concordant patterns of IPF [30]. If this cannot diagnose IPF, the next thing is to take a lung biopsy to also try to find concordant patterns of IPF [143]. It is extremely important to investigate biomarkers that may be key in diagnosing IPF. Biomarkers are objectively measured and evaluated indicators of normal physiological processes, pathological processes, or pharmacological responses to therapeutic interventions [144]. In general, biomarkers can be used for diagnosis to distinguish between diseases. They serve as a method of disease classification. They can also indicate susceptibility to a disease in healthy individuals. Prognostic biomarkers enable us to predict the disease outcome, while biomarkers linked to treatment are known as biomarkers of efficacy or treatment response [145].

**Figure 4:**
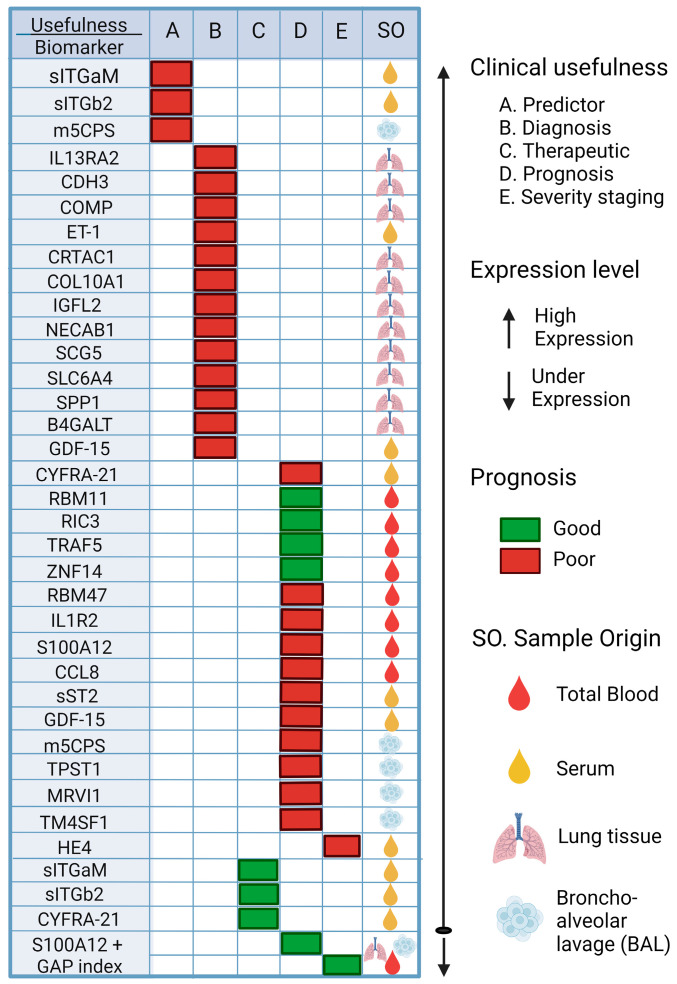
Potential biomarkers rated for Predictor, Diagnostic, Therapeutic, Prognosis, and Severity of IPF. The possible biomarkers that could be used to try to make a diagnosis in time are shown, such as the use of sITGb2 or m5CPS; if they are elevated in patients, this could indicate the appearance of FP. Other potential biomarkers are also shown to predict the prognosis of patients with FP, such as the use of RBM11, which, if overexpressed, is an indicator of a good prognosis for patients, while if RBM47 is overexpressed, it is an indicator of a poor prognosis for patients. Thus, the importance of these biomarkers becomes evident for their future validation and application to FP. sITGaM (soluble integrin subunit alpha M), sITGb2 (soluble integrin subunit beta 2), m5CPS (5-methylcytosine), IL13RA2 (interleukin 13 receptor subunit alpha 2), CDH3 (cadherin 3), COMP (cartilage oligomeric matrix protein), ET-1 (endothelin-1), CRTAC1 (cartilage acid protein 1), COL10A1 (collagen type X alpha 1), IGFL2 (like family member 2), NECAB1 (N-terminal EF-hand calcium binding protein 1), SCG5 (secretogranin V), SLC6A4 (solute carrier family 6 member 4), SPP1 (secreted phosphoprotein 1), B4GALT1 (beta-1,4-galactosyltransferase), GDF-15 (growth differentiation factor 15), CYFRA21 (cytokeratin fragment 19), RBM11 (RNA-binding motif protein 11), RIC3 (resistance to inhibitors of cholinesterase 3), TRAF5 (TNF receptor-associated factor 5), ZNF14 (zinc finger protein 14), RBM47 (RNA-binding motif protein 47), IL1R2 (interleukin 1 receptor type 2), S100A12 (S100 calcium binding protein A12), CCL8 (C-C motif chemokine ligand 8), sST2 (soluble receptor form 2), TPST1 (tyrosyl protein sulfotransferase 1), MRVI1 (inositol 1,4,5-triphosphate receptor associated 1), TMF4S1 (transmembrane 4 L six family member 1), HE4 (human epididymis protein 4) [148,149,150,151,152,153,154,155,156,157,158,159,160].

Biomarkers can be found in various specimens, such as blood, sputum, fluid, or cells from bronchoalveolar lavage (BAL) samples, and even more invasive techniques such as lung tissue biopsies [144]. Biomarkers can be very useful in IPF disease, especially for early diagnosis, and even the use of biomarkers with predictive and prognostic values, as they are indicators of disease severity or progression and can be used to guide treatment, follow-up, and survival of these patients [146,147]. Figure 4 and Table 4 describe the biomarkers that have been studied in IPF over the last two years and their potential application in IPF induced by COVID-19.

The use of these biomarkers, such as CYFRA 21-1, which can be detected in serum samples, is useful for the diagnosis and prognosis of patients with PFI; this biomarker predicted short-term progression and long-term survival (HR = 1.13 [95% CI, 0.02–1.25]; *p* = 0.023). RBM47 is another biomarker that can be assessed in peripheral blood and can be used to assess the progression of IPF. When its expression increases, a poor prognosis is assumed in patients with IPF (AUC at 4 years = 0.95).

Similarly, other biomarkers that have been studied, such as sITGaM and sITGb2, have been investigated for their predictive and therapeutic value in patients with COVID-19 with a value of R = 0.42, *p* = 0.01 [148]; high levels of these molecules have been observed in patients with COVID-19. Additionally, elevated levels of IL13RA2, CDH3, and COMP biomarkers may be useful in diagnosing IPF; the AUC for this group of three genes was reported as 0.98. Other biomarkers with prognostic value include RBM11, RIC3, TRAF5, ZNF14, and RBM47. The AUC value in the first year was low (AUC at 1 year = 0.63), but the AUC value gradually increased with time: AUC at 2 years = 0.77; AUC at 3 years = 0.85; AUC at 4 years = 0.95; this shows its predictive usefulness. The results of the survival analysis showed that high expression of RBM11, RIC3, TRAF5, and ZNF14 was associated with a good prognosis of IPF, while high expression of RBM47 was associated with a poor prognosis [154].

All of the aforementioned points underscore the necessity and utility of future research on non-invasive and efficacious biomarkers. Such molecules could offer a more holistic approach to managing patients with PF, and collectively, they have the potential to regulate and enhance the quality of life for individuals with this ailment, a condition that is likely to be further compounded by COVID-19.

## 7. Conclusions

PF is a disease that has multiple causes. Currently, treatment of the disease involves a multidisciplinary approach. The main therapies for patients with PF include oxygen therapy, pulmonary rehabilitation, and pharmacological therapy. Two FDA-approved drugs for PF are pirfenidone and nintedanib. The research landscape for PF remains broad, providing ample opportunities for researchers worldwide to expand the range of drugs and discover new therapeutic targets that can help prevent or cure PF. Nimotuzumab, an emerging drug, has shown promising results in treating inflammation and PF caused by COVID-19. Another drug, Treamid, has improved FVC and DLCO values in patients with pneumonia after COVID-19. However, it is important to note that none of these drugs can cure the disease.

Describing the molecular mechanisms involved in the pathology of PF is of great importance in order to develop new drugs or discover possible biomarkers for the correct management of both IPF and FP due to COVID-19 because currently, the diagnosis of PF is based on tomography and lung biopsy, which is an invasive method. Therefore, the incorporation of these new biomarkers, such as ET-1, which is elevated in the serum of IPF patients, cannot help in the differential diagnosis between IPF and ILD associated with autoimmune diseases (AD-ILD). Another biomarker, sST2, helps predict the deterioration of IPF patients and their outcome. Therefore, scientists worldwide must concentrate on developing new drugs that are more effective than current ones. Additionally, they must prioritize discovering and implementing predictive, diagnostic, severity classification, prognostic, and other biomarkers.

These tools enable us to predict patients’ life expectancy and facilitate comprehensive and personalized management of each patient with this disease to continue improving their quality of life.

## Figures and Tables

**Table 1:** Drugs with favorable results in the treatment of IPF and PF associated with COVID-19.

Drug	Ref.	Indication	Mechanism of Action	Phase/ID	Results or Conclusions
**Pirfenidone**	[77]	COVID-19 pneumonia	Inhibits TGF-β.	NA/NA	FEV1, FEV1%, FVC, and FVC% values were higher in the methylprednisolone group together with pirfenidone than in the methylprednisolone group.
**Nintedanib**	[75]	Fibrosis lung post-COVID-19	Tyrosine kinase inhibitor.	NA/TCTR20220426001	There was no improvement in oxygenation, chest X-ray findings, or mortality at 60 days after admission. However, the nintedanib group had a significantly greater difference in SpO_2_/FiO_2_ ratio after treatment than the controls.
**Nimotuzumab**	[76]	Inflammation and PF due to COVID-19	Modulates the dimerization of EGFR, which inhibits kinase activity.	I/II/RPCEC00000369	It is a safe antibody that could reduce IL-6 and PAI-1 and prevent fibrosis in patients with severe and moderate COVID-19 at high risk of worsening.
**Treamid**	[130]	Patients after COVID-19 pneumonia	Derived from bisamide of dicarboxylic acid; a metal chelator.	II/NCT04527354	Clinically significant improvement in FVC and/or DL_CO_ was achieved, as well as a decrease in dyspnea.
**GB0139**	[78]	COVID-19 pneumonitis	Participates in the inhibition of galectin-3.	Ib/IIa/NCT04473053 EurdraCT: 2020002230-32	It decreases circulating concentrations of galectin-3 and may also reduce inflammation.
**Collagen-Polyvinylpyrrolidone**	[79]	Hyper-inflammation of hospitalized patients with COVID-19	Downregulates pro-inflammatory cytokines, in addition to several adhesion molecules (ELAM-1, VCAM-1 and ICAM-1), as well as the expression of COX-1 and collagenase.	I/NCT04517162	Decreases IP-10, IL-8, and M-CSF and shortened the duration of symptoms; in addition, a higher mean oxygen saturation value and a higher proportion of patients retaining oxygen saturation values ≥ 92% were observed.
**Tranilast**	[80]	Severe pneumonia after COVID-19	Modulates the suppression of the expression and/or action of the TGF-β pathway.	Case report/NA	Reduced PF and improved respiratory function.
**Pycnogenol-Centellicum**	[131]	Post-COVID-19 lung disease	Pycnogenol controls and decreases edema; Centellicum, by modulating the position of collagen, slows irregular scarring, keloid scarring, and fibrosis.	NA/NA	The progression from edema to fibrosis appears to be slower or attenuated with this combination of supplements both in patients with idiopathic IPF and patients with lung disease.
**Anakinra**	[81]	PF and persistent hypoxemia in COVID-19	Antagonist of the IL-1 receptor.	Case Report/NA	Improved respiratory and clinical parameters, which allowed an early hospital discharge. During follow-up, the evolution was favorable with resolution of fibrosis.

IPF: idiopathic pulmonary fibrosis, PF: pulmonary fibrosis, FEV1: forced expiratory volume in 1 s, FEV%: total amount of air exhaled during the FEV test, FVC: forced vital capacity, FVC%: percentage forced vital capacity, SpO_2_/FiO_2_: ratio of peripheral arterial oxygen saturation to the inspired fraction of oxygen, DL_CO_: diffusing capacity for carbon monoxide, TGF-β: transforming growth factor β, IP-10: small-inducible cytokine B10, IL-8: interleukin-8, M-CSF: macrophage colony-stimulating factor, ELAM-1: endothelial leukocyte adhesion molecule-1, VCAM-1: vascular cellular adhesion molecule-1, ICAM-1: intercellular adhesion molecule 1, COX-1: cyclooxygenase 1, IL-1: interleukin-1, NA: not available.

**Table 2:** Molecules for the treatment of IPF and their potential use in PF due to COVID-19.

Drug	Ref.	Indication	Mechanism of Action	Phase	Results or Conclusions
**Pamrevlumab (FG-3019)**	[136]	IPF	Modulates the inhibition of action of connective tissue growth factor (CTGF).	II	Pamrevlumab attenuated the progression of IPF and was well tolerated, reducing the decrease in the predicted FVC percentage by 60; 3% at week 48.
**EW-7197**	[132]	IPF	Blocks the catalytic activity of ALK5 by competitively blocking ATP from binding to ALK5.	Preclinical	In fibrosis, inhibited TGF-β-/Smad2/3 and ROS signaling.
**ZSP1603**	[137]	PF	Blocks PDGF-Rβ and ERK. It also inhibited primary human PF differentiation by reducing the expression of TGF-β1, TIMP-1, and COL1A1.	Preclinical	Significantly attenuated lung damage, inflammation, and fibrosis in vivo in an animal model.
**Piceatannol**	[135]	Possible use in PF	An analogue of the polyphenolic compounds of resveratrol that inhibits non-receptor tyrosine kinase-Syk.	Preclinical	In an in vitro model, it modulates fibroblast activation induced by the TGF-β1 pathway and ECM production.
**Alamandine**	[133,138]	PF	A peptide with protective effects on the cardiovascular system, in addition to vasodilator and antifibrotic effects.	Preclinical	Attenuated collagen deposition and improved ventilatory mechanics in PF in a Wistar rat model.
**Thymol**	[139]	PF	Participates in modulating oxidative stress, inflammation, expression of miR-29a/TGF-β, and PI3K/phospho-Akt signaling in PF.	Preclinical	Fibrotic markers were reduced: α-SMA and fibronectin, inflammatory mediators; TNF-α, IL-1β, IL-6, and NF-κB and biomarkers of oxidative stress; MDA, GSH, and SOD. Prevented bleomycin-induced PF.
**Esomeprazole and pirfenidone**	[140]	PF	Esomeprazole has anti-inflammatory and antifibrotic activity.Pirfenidona inhibits TGF-β.	Preclinical	Mixing esomeprazole with pirfenidone improves the antifibrotic efficacy of pirfenidone.
**AT13387**	[134]	Chronic lung injury and PF	Heat shock protein inhibitor 90.	Preclinical	Treatment with AT13387 15 mg/kg reduced alveolar inflammation, fibrosis, and NLRP3 staining and blocked activation of ERK and HSP90. It also reduces collagen deposition, chronic lung injury, and airway hyper-responsiveness.
**GSK3335103**	[141]	Fibrotic disease	A novel inhibitor of integrin αvβ6 mimetic of RGD.	In vitro and preclinical	Attenuates TGF-β signaling in vitro and in vivo with a well-defined pharmacokinetic/pharmacodynamic relationship. That is, it reduces collagen deposition in vivo.
**Diethylcarbamazine**	[142]	Antiparasitic and antifibrotic	Immunomodulatory, anti-inflammatory, and antifibrotic activities.	NA	Still without results; however, reduces the production of fibrotic factors and collagen.

IPF: idiopathic pulmonary fibrosis, PF: pulmonary fibrosis, CTGF: connective tissue growth factor, FVC: forced vital capacity, ALK5: TGF-β type I receptor kinase, ATP: adenosine triphosphate, ROS: reactive oxygen species, PDGF-Rβ: PDGF receptor-beta, ERK: extracellular signal-regulated kinase. TIMP1: tissue inhibitor of matrix metalloprotease 1, COL1A1: collagen type I alpha 1, ECM: extracellular matrix, TNF-α: tumor necrosis factor alpha, IL-1β: interleukin-1β, IL-6: interleukin-6, NF-κB: nuclear factor kappa-light-chain-enhancer of activated B cells, MDA: malondialdehyde, GSH: reduced glutathione, SOD: superoxide dismutase, HSP90: heat shock protein 90, RGD: arginine-glycine-aspartic acid peptide, NA: not available.

**Table 3:** Studies registered on ClinicalTrials.gov in order to evaluate molecules for the treatment of PF and its possible use in lung damage caused by COVID-19.

Title	Status	Conditions	Molecule	Phase	NTC Number (Accessed November 2023)
Pirfenidone compared to placebo in PF post-COVID-19	Recruiting	PF post-COVID-19 infection	Pirfenidone	Phase 2	NCT04607928
Colchicine and PF post-COVID-19	Active, not recruiting	COVID-19 IPF	Colchicine	Phase 4	NCT04818489
Treatment of PF due to COVID-19 with Fuzheng Huayu	Completed	PF due to COVID-19	Drug: Fuzheng Huayu Tablet Drug: Vitamin C tablets	Phase 2	NCT04279197
Safety and effectiveness of EV Pure + WJ-Pure Treatment on PF secondary to COVID-19	Recruiting	PF COVID-19 Respiratory infection	Drug: EV-Pure™ and WJ-Pure™ plus standard care	Phase 1	NCT05387239
Anti-inflammatory and anti-fibrotic drugs in PF post- COVID-19	Completed	Post-COVID-19 syndrome Lung fibrosis	Drug: Corticosteroids alone Drug: Corticosteroids + Colchicine Drug: Corticosteroids + Pirfenidone Drug: Corticosteroids + Colchicine + Pirfenidone	Not Applicable	NCT05648734
Short term low dose corticosteroids for management of PF post-COVID-19	Completed	COVID-19	Drug: 20 Mg Prednisone for 14 days	Not Applicable	NCT04551781
Mineralocorticoid receptor antagonist and PF in COVID-19	Recruiting	COVID-19	Drug: Canrenoate potassium	Phase 4	NCT04912011
Efficacy and safety of nintedanib in the treatment of PF in patients with moderate to severe COVID-19	Unknown status	COVID-19	Nintedanib	Phase 2	NCT04338802
Detection of integrin avb6 in IPF, PSC, and COVID-19 using PET/CT	Recruiting	IPF Primary sclerosing cholangitis COVID-19 pneumonia	Drug: FP-R01-MG-F2	Early Phase 1	NCT03183570
Intramuscular effect of polymerized type I collagen on the cytokine storm in COVID-19 patients	Recruiting	COVID-19 Cytokine storm Regulation of inflammatory response PF	Collagen-Polyvinylpyrrolidone	Phase 1 Phase 2	NCT04517162
Assessing the efficacy of sirolimus in patients with COVID-19 pneumonia for prevention of post-COVID fibrosis	Recruiting	PF COVID-19 pneumonia Long COVID	Drug: Sirolimus	Phase 2 Phase 3	NCT04948203
Study to assess efficacy and safety of treamid for patients with reduced exercise tolerance after COVID-19	Not yet recruiting	SARS-CoV-2 infection Lung fibrosis	Drug: Treamid	Phase 2 Phase 3	NCT05516550
The MONACO cell therapy study: monocytes as an antifibrotic treatment after COVID-19	Recruiting	PF ILD COVID-19	Biological: MON002	Phase 1 Phase 2	NCT04805086
Study of longidaze in the prevention and treatment of PF, interstitial lung disease caused by COVID-19	Recruiting	PF	Drug: Bovhyaluronidase azoxymer	Not Applicable	NCT04645368
Safety and effectiveness of cyclosporin in the management of COVID-19 ARDS patients in Alexandria University Hospital	Not yet recruiting	COVID-19 acute respiratory distress syndrome Cytokine release syndrome PF	Drug: Cyclosporine	Phase 3	NCT04979884
BIO 300 oral suspension in previously hospitalized long COVID-19 patients	Recruiting	COVID-19 Long COVID PF ARDS Post-acute respiratory complications of COVID-19	Drug: BIO 300 Oral Suspension	Phase 2	NCT04482595
The study of the use of nintedanib in slowing lung disease in patients with fibrotic or non-fibrotic interstitial lung disease related to COVID-19	Recruiting	PF Interstitial lung disease Respiratory disease	Drug: Nintedanib	Phase 4	NCT04619680
Pirfenidone vs. nintedanib for fibrotic lung disease after coronavirus disease-19 pneumonia	Active, not recruiting	Novel coronavirus-induced lung fibrosis	Drug: Pirfenidone Drug: Nintedanib	Phase 4	NCT04856111
Pilot study to assess efficacy and safety of Treamid in the rehabilitation of patients after COVID-19 pneumonia	Completed	SARS-CoV-2 infection PF	Drug: Treamid	Phase 2	NCT04527354
Use of cSVF via IV deployment for residual lung damage after symptomatic COVID-19 infection	Enrolling by invitation	Pulmonary alveolar proteinosis COPD IPF Viral pneumonia Coronavirus infection ILD	Procedure: Microcannula harvest adipose derived tissue stromal vascular fraction (tSVF) Device: Centricyte 1000 Procedure: IV deployment of cSVF in sterile normal saline IV solution Drug: Liberase Enzyme (Roche)	Early Phase 1	NCT04326036
APX-115 use in hospitalized patients with confirmed mild to moderate COVID-19	Recruiting	COVID-19	Drug: APX-115	Phase 2	NCT04880109

**Table 4:** Potential Biomarkers in IPF and their possible use in PF associated with COVID-19.

Biomarker and Matrix	Ref.	Biological Function	Type of Biomarker	Findings
**sITGaM and sITGb2** **Samples of serum**	[148]	Integrins participate in the inflammatory response by contributing to the anchoring of various cells, such as leukocytes, to the endothelium, thus allowing their diapedesis.	Predictor Therapeutic	Elevation of soluble integrin subunits was reported in the (+) group; therefore, they may be biomarkers for predicting pulmonary complications and, thus, a potential therapeutic target in post-COVID-19 patients. R = 0.42, *p* = 0.01.
**IL13RA2, CDH3 and COMP** **Lung tissue**	[149]	IL-13Rα2: acts as a non-signaling decoy receptor. CDH3: a classic cell-to-cell adhesion molecule; regulates multiple cellular homeostatic processes in normal tissue. COMP: an ECM glycoprotein; participates in fibrillogenesis and collagen secretion.	Diagnostic	Elevation of IL13RA2, CDH3, and COMP could serve as a diagnostic signature for IPF and could offer new insights into the underlying diagnosis of IPF. The area under the curve reported for the three-gene group was 0.98.
**Endothelin-1** (**ET-1**)**.** **Samples of serum**	[150]	A molecule produced by the vascular endothelium, which is involved in the homeostasis of vascular tone.	Diagnostic	Serum levels of ET-1 were found to be elevated, so it may be useful as a biomarker of PID, but it could not help in the differential diagnosis between IPF and ILD associated with autoimmune diseases (AD-ILD). In addition, ET-1 levels may be associated with the severity of ILD. The area under the curve reported was 0.803 (95% CI: 0.728–0.878).
**Calcium binding protein S100 A12.** **Lung tissue,** **Blood and** **Bronchoalveolar lavage.**	[151]	S100A12 is involved in the adhesion and migration of leukocytes and in the production of cytokines and chemokines.	Prognosis/Severity	Down-expression of S100 A12 and the composite variable may be a more effective predictive index.
**CYFRA 21-1** **Samples of serum.**	[152,153]	Keratins are part of the cytoskeleton of epithelial cells. Cytokeratin-19 is expressed by airway epithelial cells.	Diagnostic and Prognosis Therapeutic	It predicted short-term progression and long-term survival when assessed cross-sectionally and at serial time points, notably beyond 3 months. Because it is a marker of epithelial damage and turnover, it may have potential utility as a prognostic and therapeutic biomarker in people with IPF. CYFRA 21-1 demonstrated an association with overall mortality in both the discovery (HR, 1.13 [95% CI, 0.02–1.25]; *p* = 0.023) and validation cohorts (HR = 1.12 [95% CI, 0.02–1.25]; *p* = 0.023) 1.06–1.19]; *p* = 0.0001).
**RBM11, RIC3, TRAF5, ZNF14 and RBM47. Related genes with m6A.** **Peripheral blood**	[154]	TRAF5: an important signal transducer for a wide range of TNF receptor superfamily members, and it mainly mediates the activation of NF-κB pathway. RBM47: involved in EMT. RIC3: a chaperone protein. RBM11: involved in cellular response to oxidative stress and regulation of alternative splicing. ZNF14: has a zinc finger and a Kruppel-associated box (KRAB) domain. This domain is involved in the transcriptional repression of several zinc finger proteins.	Prognosis	Survival analysis showed that high expression of RBM11, RIC3, TRAF5, and ZNF14 was associated with a good prognosis of IPF, while high expression of RBM47 was associated with a poor prognosis. The AUC value in the first year was low (AUC at 1 year = 0.63), and the AUC value gradually increased with time (AUC at 2 years = 0.77, AUC at 3 years = 0.85, AUC at 4 years = 0.95); this shows the prediction utility.
**CRTAC1, COL10A1, COMP, IGFL2, NECAB1, SCG5, SLC6A4 and SPP1.** **Lung tissue**	[154]	COMP: a non-collagenous glycoprotein component of extracellular matrix (ECM) that accentuates TGF-β1 signaling and is associated with extracellular matrix polymerization and stiffness. SPP1: a protein formerly related to PF and COPD in lung process in mice. SLC6A4: a serotonin transporter gene. COL10A1: members of the collagen family. CRTAC1: regarded as an opponent of nogo receptor-1. IGFL2, NECAB1, SCG5: found to play a role in PF.	Diagnostic	These genes were expressed at high levels and associated with monocytes, plasma cells, neutrophils, and regulatory T cells (T reg), suggesting their use as diagnostic biomarkers of IPF. Their diagnostic ability showed an advantageous diagnostic value, with an AUC of 0.943 (95% CI 0.883–0.986) in CRTAC1, AUC of 0.886 (95% CI 0.778–0.970) in COL10A1, AUC of 0.984 (95% CI 0.956–1.000) in COMP, AUC of 0.633 (95% CI 0.469–0.782) in RPS4Y1, AUC of 0.936 (95% CI 0.873–0.980) in IGFL2, AUC of 0.925 (95% CI 0.841–0.987) in NECAB1, AUC of 0.967 (95% CI 0.923–0.995) in SCG5, AUC of 0.763 (95% CI 0.625–0.882) in SLC6A4, and AUC of 0.886 (95% CI 0.790–0.957) in SPP1.
**B4-galactosyltransferase** (**B4GALT1**)**.** **Lung tissue**	[155]	B4GALTs are involved in the expression of biologically active carbohydrates through glycosylated glycans.	Diagnostic	High levels of B4GALT1, both in mRNA and protein, were found in 4 patients with IPF and in the primary human cells derived from IPF. A positive correlation was found between B4GALT and genes belonging to the EMT pathway (*p* = 0.01).
**IL1R2, S100A12 and CCL8.** **Peripheral blood**	[156]	IL1R2: through competitive binding with IL-1β, prevents it from binding to IL1R1, blocking the signal transduction of IL-1β in inflammatory diseases and acting as a bait receptor. S100A12: has a proinflammatory cytokine function; is related to the fibrotic process of skin scars. CCL8: expressed by monocytes/macrophages in inflammatory tissues, stimulated by T cells and accessory pathways of IFN and other pro-inflammatory cytokines or by innate mechanisms.	Prognosis	Increased expression of IL1R2, S100A12, and CCL8 could predict survival at 1, 2, and 3 years. The AUCs reported for predicting 1-, 2-, and 3-year survival were greater than 0.7.
**sST2** **Serum**	[157]	ST2: belongs to the interleukin-1 (IL-1) receptor family, which exists in transmembrane (ST2L) and soluble (sST2) isoforms.	Prognosis	High levels of serum sST2 have been found in patients with PF, and this level helped predict greater deterioration and poorer outcomes in these patients. The overexpression of sST2 increased the hazard ratio to 1.005 (95% CI: 1.001–1.010).
**Growth differentiation factor-15** (**GDF-15**)**.** **Serum**	[158]	Also known as macrophage inhibitory cytokine-1 (MIC-1) and is a member of the TGF-β family.	Diagnostic and Prognosis	Elevated expression of GDF-15 could be a promising biomarker for the occurrence and survival of acute exacerbations (AEs) in patients with IPF. The areas under the ROC curves of serum GDF-15 levels in patients with AE-IPF or S-IPF showed significance (ROC: 0.738, *p* < 0.001, 95% CI: 0.529–0.809, cut-off value 989.3 pg/mL).
**m5CPS** **Bronchoalveolar lavage**	[159]	Affects a series of biological functions, such as RNA stabilization and translation.	Prediction and Prognosis	Expression of m5CPS was positively associated with active mast cell infiltration levels in all training, testing, and validation cohorts. Therefore, the results suggest important roles of mast cells in IPF. In terms of the results, m5CPS could predict the 1-, 3-, and 5-year survival rates of IPF patients with high accuracy (AUC = 0.803–0.973).
**Human epididymis protein 4** (**HE4**)**.** **Serum**	[160]	The epididymis-specific protein can be found in other tissues, such as the respiratory tract, and its main function is to inhibit the activity of several proteases, such as MMP2 and MMP9, which contribute to the progression of fibrosis.	Severity and prognosis	High levels of HE4 were found in patients with IPF, especially in patients with AE-IPF, and in statistical analysis, serum levels of HE4 [hazard ratio (HR) = 1.004, *p* = 0.042] and GAP index (HR = 1.374, *p* = 0.010) were associated with worse survival in patients with IPF.
**TPST1, MRVI1 and TM4SF1** **Bronchoalveolar lavage**	[161]	TPST1: a member of a family of sulfotransferase proteins, involved in the sulfidation of tyrosine residues. MRVI1: the cGMP kinase substrate associated with the inositol 1,4,5-trisphosphate receptor; it participates in the regulation of intracellular calcium induced by IP3 through a NO/PRKG1-dependent mechanism. TM4SF1: a tumor-associated protein that is widely expressed in multiple human cancers, is localized at the surface of the cell membrane and late endocytic organelles, and plays a vital role in cell motility.	Prognosis	The discharge expression of these biomarkers showed that inflammation and immunological processes significantly affected the prognosis of IPF patients. The areas under the curve reported for 1-, 2-, and 3-year survival rates were 0.862, 0.885, and 0.833 (TPST1, MRVI1, and TM4SF1), respectively.
